# Lichen planus following recombinant zoster vaccine

**DOI:** 10.1016/j.jdcr.2025.09.002

**Published:** 2025-09-18

**Authors:** Charli C. Gruen, Bianca N. Coffel, Paul Hahn

**Affiliations:** a F. Edward Hébert School of Medicine, Uniformed Services University of the Health Sciences, Bethesda, Maryland; b Department of Dermatology, San Antonio Uniformed Services Health Education Consortium, San Antonio, Texas

**Keywords:** lichen planus, recombinant zoster vaccine, shingles, SHINGRIX, vaccination, varicella-zoster virus

## Introduction

Vaccine-related cutaneous reactions are well-documented in dermatology, with varying presentations depending on the type of vaccine and the patient’s immune response. Shingrix, a nonlive recombinant herpes zoster vaccine approved for use in the United States since 2017, is generally well tolerated but has been associated with localized and, rarely, systemic cutaneous reactions.^1^, ^2^, ^3^ Lichen planus (LP) and lichenoid drug eruptions (LDEs) can arise as a response to various triggers, including medications, infections, and vaccinations. LP has infrequently been reported after administration of vaccines including hepatitis B, influenza, tetanus-diphtheria-acellular pertussis, COVID-19, and the live attenuated zoster vaccine that was discontinued in the United States in 2020 (Zostavax).^4^, ^5^, ^6^, ^7^ While one case of LP was recently reported following both Shingrix and COVID-19 vaccines, literature on LP associated with Shingrix alone is limited.^3^ Here, we provide a new report of LP following singular administration of the recombinant zoster vaccine series and briefly discuss the clinical presentation, possible pathogenesis, and treatment of this uncommon observation.

## Case report

A 50-year-old female with Fitzpatrick skin type III presented with a rash present for 5 months, appearing 1 month after her first Shingrix vaccine dose. Initially, she noticed a single, pruritic lesion on her right middle back and several blisters on her bilateral ankles and feet that improved without treatment. Following the second Shingrix dose 4 months later, a widespread, severely pruritic rash erupted on her torso, upper, and lower extremities. Her primary care provider prescribed bacitracin zinc 500 units/g topical ointment with no improvement, then triamcinolone topical cream 0.025% and hydroxyzine 10 mg oral tablets, which provided some relief. She denied any history of similar reactions or recent changes.

**Fig 1:**
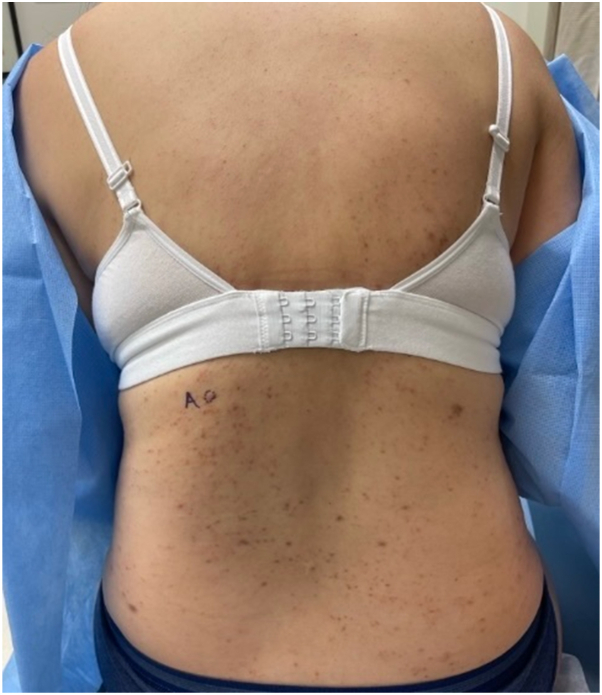
Numerous erythematous, violaceous, and skin-colored papules on the torso. Some lesions have evidence of overlying erosion.

**Fig 2:**
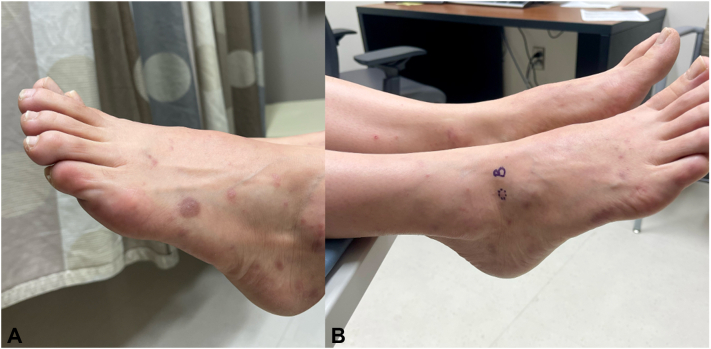
**A,** Numerous violaceous flat papules and smooth plaques with evidence of isomorphic response on the dorsal feet. Erythematous papules with central erosion on the shins bilaterally. **B,** A biopsied lesion on the right lateral foot demonstrated a well-circumscribed violaceous flat papule.

Physical examination revealed numerous violaceous flat papules and smooth plaques with isomorphic response on the dorsal feet and right wrist, erythematous papules with central erosion on the shins bilaterally, and numerous erythematous, violaceous, and skin-colored papules on the torso and buttocks (Figs 1 and 2). No nail or mucosal involvement was initially observed, and the general physical exam was otherwise unremarkable.

Skin biopsies from her left middle back and right lateral foot were consistent with lichen planus, showing characteristic lichenoid inflammation associated with necrotic keratinocytes, rete ridges with a sawtooth configuration, and fibrosis and pigmented macrophages in the papillary dermis. The patient was prescribed 0.1% triamcinolone ointment to be applied twice daily Monday through Friday for 1 month.

At 1-month follow-up, she reported significant improvement in cutaneous symptoms, and physical exam revealed resolving papular lesions and postinflammatory hyperpigmentation. At 2-months, new white lacelike reticulations appeared in her buccal mucosa after a viral illness. She was prescribed an oral suspension and triamcinolone 0.1% mucosal paste.

## Discussion

This case highlights a rare association between the recombinant zoster vaccine (Shingrix) and the development of LP, a chronic inflammatory dermatosis characterized by violaceous papules, plaques, and mucosal involvement. While one recent case report described LP following the administration of *both* Shingrix and a COVID-19 vaccine, to our knowledge, this represents the first well-documented case of LP developing secondary to the Shingrix vaccine series *alone*. A search of the CDC’s Vaccine Adverse Event Reporting System revealed 7 reported cases of LP potentially linked to Shingrix administration as of May 2025, indicating that this association remains uncommon.^8^

The patient’s clinical presentation and histopathologic findings are consistent with a diagnosis of LP, and the temporal association between the onset of symptoms following the first Shingrix dose and significant exacerbation and spread after the second dose strongly suggests a vaccine-induced etiology. The pathogenesis of vaccine-induced LP remains incompletely understood but may involve molecular mimicry or an adjuvant effect stimulating a T-cell mediated autoimmune response against keratinocytes.^5^^,^^7^ Although Shingrix is a recombinant subunit vaccine rather than a live attenuated virus, it contains glycoprotein E, which contributes to immune regulation in the setting of viral pathogenesis and vaccine efficacy, and an adjuvant component that could possibly trigger immune dysregulation in susceptible individuals.^9^

Management of vaccine-induced LP involves addressing both cutaneous and mucosal symptoms. Topical corticosteroids are first-line treatments and were effective in improving the patient’s symptoms; however, the subsequent development of mucosal involvement necessitated additional therapy with corticosteroid mucosal paste and compounded mouthwash. While the patient experienced significant improvement in cutaneous lesions, the mucosal involvement underscores the chronic and relapsing nature of LP, which may require long-term management and follow-up.

## Conflicts of interest

None disclosed.
